# The participation of Early Maladaptive Schemas (EMSs) in the perception of pain in patients with migraine: A psychological profile

**DOI:** 10.1590/1980-57642018dn12-010010

**Published:** 2018

**Authors:** Ketlin Helenise dos Santos Ribas, Valdenilson Ribeiro Ribas, Silano Souto Mendes Barros, Valéria Ribeiro Ribas, Maria da Glória Nogueira Filizola, Renata de Melo Guerra Ribas, Paulo César da Silva, Carlos Augusto Cardoso Kucera, Hugo André de Lima Martins

**Affiliations:** 1 Universidade Federal de Pernambuco, Mestrado em Neuropsiquiatria e Ciências do Comportamento Recife, PE, Brazil; 2 Universidade Federal de Pernambuco, Doutorado em Neuropsiquiatria e Ciências do Comportamento Recife, PE, Brazil; 3 Universidade Federal de Pernambuco, Fisioterapia, Recife, PE, Brazil; 4 Cérebro e Tecnologia Neurofeedback Recife (CTNR) - Cursos/Pesquisas Jaboatão dos Guararapes, PE, Brazil

**Keywords:** early maladaptive schemas, migraine, psychological profile, Esquemas iniciais desadaptativos, migrânea, perfil psicológico

## Abstract

Young's early maladaptive schemas questionnaire (YSQ-S3) is used to understand psychological aspects.

**Objective:**

EMSs were evaluated in patients with migraine.

**Methods:**

Sixty-five subjects were evaluated using the YSQ-S3 under standard conditions in a room with air conditioning at 22 ± 2°C. The subjects were stratified by morbidity (migraine), gender (male/female) and age (18-29 / 30-39 / 40-55). Controls (without migraine), n = 27 and patients (with migraine), n = 38, men (n = 19) and women (n = 46); participants aged 18-29 years, n = 34, aged 30-39 years, n = 14 and aged 40-55 years, n = 17. Data were analyzed using the Chi-square test, with p-values <0.05. Results were expressed as percentages in contingency tables.

**Results:**

There was a significant association between migraine and female gender (84.21%; p-value <0.05, Table 1), between hypervigilance and inhibition, and unrelenting standards (56.52%; p-value <0.0.014, Table 2) and female gender with migraine. Moreover, there was a significant association between hypervigilance and inhibition, and unrelenting standards (73.68%; p-value <0.0001) and self-punishment (84.21%; p-value <0.0001) in patients with migraine of both genders (Table 3).

**Conclusion:**

The individuals with migraine had a psychological profile of being overly demanding with themselves and others and self-punishing, where this was more frequent in women.

## RESUMO

O questionário de esquemas iniciais desadaptativos de Young (YSQ-S3) é usado para se entender aspectos psicológicos.

**Objetivo:**

Os EIDs foram avaliados em pacientes com migrânea.

**Métodos:**

Sessenta e cinco indivíduos foram avaliados utilizando YSQ-S3 sob condições padrão em uma sala com ar condicionado a 22 ± 2°C. Os sujeitos foram estratificados pela morbidade (migrânea), por gênero (masculino/feminino) e por idade (18-29 / 30-39 / 40-55). Controle (sem migrânea), n = 27 e com migrânea, n = 38, homens (n ​​= 19) e mulheres (n = 46); indivíduos com idade entre 18 a 29 anos, n = 34, entre 30-39 anos, n = 14 e entre 40-55 anos, n = 17. Os dados foram analisados ​​usando o teste do qui-quadrado, com valores p <0,05. Os resultados foram expressos em porcentagens em tabelas de contingência.

**Resultados:**

Houve associação significativa entre migrânea e gênero feminino (84,21%; p-valor <0,05, Tabela 1), entre hipervigilância e inibição, e padrões excessivamente rígidos ​​(56,52%; p-valor <0,0,014, Tabela 2) e gênero feminino com migrânea. Além disso, houve associação significativa entre hipervigilância e inibição, e padrões excessivamente rígidos ​​(73,68%; p-valor <0,0001) e autopunição (84,21%; p-valor <0,0001) em pacientes com migrânea de ambos os gêneros (Tabela 3).

**Conclusão:**

Os indivíduos com migrânea têm um perfil psicológico de serem excessivamente exigentes consigo e com os outros e de se autopunirem, sendo isso mais frequente em mulheres.

One of the main goals of the physiotherapist is to alleviate or eradicate acute and chronic pain in patients.^1^ However, even with the proper use of several techniques, the different forms of expression of pain among individuals^2^ still appear to pose a great challenge in the interpretation of the efficacy of the treatment process, hindering even the perception of its termination.^3^ From the physiological point of view, knowledge about the difference between the peripheral and central circuits of pain seems only to provide support to understand the differences between the perceptions of acute and chronic pain, respectively.^4^ Substances such as bradykinin, prostaglandins, leukotrienes, substance P, serotonin and acetylcholine are released with peripheral pain. These substances act on different populations of neurons, reducing the activation threshold of nociceptors.^4^^,^ 5 In central sensitization, the responses of dorsal horn neurons are increased after repeated stimulation of C-fibers, which release glutamate and stimulate N-methyl-D-aspartate (NMDA) receptors in these neurons until reaching the thalamus.^5^^,^ 6

Understanding of the pathophysiologic mechanism of pain does not yet seem to explain the diverse responses of different patients to similar intensities of pain that follow the same circuit. One individual with acute pain shows little evidence of that pain^7^ while another subject demonstrates the sensation of pain by much suffering^8^ expressed through resounding cries.^9^

Thus, the major problem for the physiotherapist still seems to involve the different behaviors related to pain intensity.^2^ There are descriptions in the literature^10^^,^ 11 concerned with the cognitive model of fear arising from the need to avoid pain.^12^ However, the impression is that this perspective does not yet answer the question of how to help patients minimize their suffering. In this context, it seems relevant to understand the origin of the different expressions of sensations, which often leads to the physiotherapist feeling a sense of failure after several tiresome maneuvers during the recovery process.^9^

Thus, for this study we chose an interdisciplinary approach to understand the psychological aspects of pain, not for treatment as such, but to see the patient as a whole and be able to refer them for treatment of the psyche, if their needs no longer relate to the neurophysiological circuits of pain perception. In this interdisciplinary literary search, Young (2003)^13^ appears to give more support to this approach. He establishes that people internalize thoughts in childhood, which become part of their personality structure and that, in adulthood, these thoughts can promote social adaptation or maladjustment.^14^

For thought to exist, it is necessary to have fully functioning neural networks,^15^ with each neural network accommodating a type of thinking that is modulated by small chains of proteins called peptides.^16^^,^ 17 Thus, the way of thinking and acting in the world mobilizes neurochemical signaling, because, for each type of thinking, the hypothalamus releases neuropeptides that enable short-term or long-term mobile synapses.^18^

The activation of these neural networks in schematic formats involves the ways of thinking and acting as human beings; Young (2003)^19^ called this complex set ´early schemas´ and according to him, they can be adaptive or maladaptive. After many years of studies, Young constructed a scale to identify the early maladaptive schemas (EMSs) in subjects^19^ that can be modified in psychotherapy sessions.^20^

Thus, the purpose of this study was to investigate the participation of EMSs in the perception of pain, especially migraine, because this is not merely a headache, but an active and incapacitating disease classified by the Brazilian Society of Headache and also because it is a risk factor for brain lesions.^21^

## METHODS

### Subjects

Sixty-five patients, 38 with migraine (Migraine Group) and 27 without migraine (Control Group) from a population of 207 patients of the Brain and Neurofeedback Technology (Clinic/Cérebro e Tecnologia Neurofeedback Recife) - CTNR were evaluated. These patients were undergoing brain training at the CTNR and presented at the clinic with medical diagnoses indicative for this auxiliary neurofeedback treatment. The patients studied were university students, air force personnel and businesspersons. Only the university students were single, while all the other subjects were married. All subjects were submitted to evaluations using the EMS questionnaire (YSQ-S3) under standard conditions in an air-conditioned room at a temperature of 22 ± 2°C. Inclusion criteria were subjects of both sexes aged 18-60 years, who had chronic migraine without aura and used non-opioid analgesics, antiemetic agents or anti-inflammatory drugs only during crises. Chronic migraine without aura was defined according to the second and revised edition of the International Headache Society classification of 2004,redefined to include chronic headache occurring on eight (previously 15) or more days per month for more than three months in the absence of overuse of medications. Subjects under 18 or over 60 years of age were excluded according to the criterion of the YSQ-S3 validation process in Portuguese.^22^

**Groups**

The Chi-Square Distribution and Simple Random Method (90% confidence level with 10% probability of error) were used.^23^

$n=\frac{\chi^{2}*N*P*Q}{e^{2}*\left(N-1\right)+\chi^{2}*P*Q}$

- n = sample size to be calculated;
- *χ* ^2^ = Chi-Square (3.8416);
- α = 10% = 0.1 =>Level of Significance with (1 - α) = 90% = 0.9 => Confidence level;
- N = Total number of patients (207);
- P = 0.5 = 50% success;
- Q = 0.5 = 50% failure;
- e^2^ = 0.1 = 10% (Sampling error).

Therefore:

$n=\frac{3.8416*207-0.5*0.5}{{\left(0.1\right)}^{2}*\left(206\right)+3.8416*0.5*0.5}=\frac{198.80}{2.06+0.9604}=\frac{198.80}{3.0204}=65,81≅65\;\mathrm{patients}$

The subjects of this cross-sectional study were stratified in two ways (gender and age group):

*Stratification by Gender*

Two subgroups were formed from the 65 patients: male (n = 19: Control = 6; Migraine = 13) and female (n = 46: Control = 14; Migraine = 32).

*Stratification by age*

Three subgroups were formed from the 65 patients: individuals aged 18-29 years (n = 34), 30-39 years (n = 14) and 40-60 years (n = 17).

### Assessments

This work was approved by the local Research Ethics Committee (CAAE Number #1.383.600 on January 5, 2016). Before data collection, all the subjects signed informed consent forms and all the women ensured that they were not in the pre-menstrual period and in the prodromal period of the migraine on the day of the assessment.

### Specifications of the Young Schema Questionnaire - Short Form 3 (YSQ-S3)

The EMS questionnaire used in this study was developed by Jeffrey Young in 2003^19^ and adapted to Portuguese by Pinto Gouveia et al. in 2005.^22^ The 90-question version is based on research on absent paternal and/or maternal figures or their failures during early life education, a phase that enables adaptation. Young found in his studies that if there is a lack of basic security in the family and in the group, including related to problems of attachment, autonomy, self-esteem, self-expression and real limits, the individual can acquire EMSs that may lead to a personality disorder.^24^

The Portuguese version of the YSQ-S3 22 is composed of 90 questions using a Likert scale ranging from 1 to 6:^25^

- 1 = completely false, that is, has definitely nothing to do with what happens to me;
- 2 = false most of the time, that is, it has almost nothing to do with what happens to me;
- 3 = a little truer than false, that is, it has a little to do with what happens to me;
- 4 = moderately true, that is, in some way it has something to do with what happens to me;
- 5 = true most of the time, that is, it has a lot to do with what happens to me;
- 6 = describes me perfectly, that is, it has everything to do with what happens to me.

Young identified 18 EMSs organized in five domains (disconnection/rejection, impaired autonomy, impaired limits, orientation to the other, and hypervigilance and inhibition),^19^ which, when impaired, lead to dysfunctional schemas, as described in the following table:

**Table:** 

Domain	Early maladaptive schemas	Origin / Etiology
**1^st^ Domain:** Disconnection and rejection	• Fear of abandonment • Mistrust • Emotional deprivation • Defectiveness/shame • Social isolation/alienation	Linked to failures of secure attachment to others, of affection and stability, of mothering in general. Serious difficulties in establishing healthy affective relationships.
**2^nd^ Domain:** Impaired autonomy and performance	• Dependency/incompetency • Vulnerability to harm or illness • Enmeshment • Failure	Individuals cannot develop a sense of confidence, of establishing themselves in the world by themselves, generally possessing overprotective families that, in an attempt to protect the child, end up not reinforcing their autonomy.
**3^rd^ Domain:** Impaired limits	• Entitlement/grandiosity • Insufficient self-discipline	Linked to failures to apply realistic limits, the ability to follow rules and norms, respect the rights of others and fulfill personal goals. Selfishness is the main characteristic of these individuals, and the family is generally permissive.
**4^th^ Domain:** Orientation to the other	• Subjugation • Self-sacrifice • Recognition-seeking	In order to gain approval and avoid retaliation, patients in this domain have an overemphasis on meeting the other's wants and needs at the expense of their own needs. The family of origin usually establishes a conditional love relationship, that is, the child only receives attention and approval, if it suppresses its free expression and behaves in the desired way.
**5^th^ Domain:** Hypervigilance and inhibition	• Negativism/pessimism • Emotional inhibition • Unrelenting standards • Punitiveness	Because of a rigid, repressive education in which there was no possibility to express their emotions in a free way, individuals with schemas linked to this domain are generally sad and introverted, with overly rigid internalized rules, exaggerated self-control and pessimism, and hypervigilance for possible negative events.

### Data analysis

Data analysis was carried out using the SIGMA STAT computer program for Windows - Version 2.0 (Jandel Corporation). The results were analyzed using the Chi-square test, with a p-value <0.05 considered statistically significant. Results are expressed as percentages and represented in contingency tables.

## RESULTS

### Association between migraine, gender and age group

There was a statistically significant association between migraine and female gender (84.21%; p-value <0.05 - Table 1).

**Table 1:** Association between morbidity, gender and age group.

Variable		Group					p-value Chi-square
		Control			Migraine		
		n	%		n	%	
**Total**		**27/65**			**38/65**		
Gender	Male	13/27	48.14		6/38	15.78	0.018*
	Female	14/27	51.85		32/38	84.21	
Age (years)	18-29	13/27	48.14		21/38	55.26	0.868
	30-39	5/27	18.52		9/38	23.68	
	40-55	9/27	33.33		8/38	21.5	

* Statistically significant.

### Association of hypervigilance and inhibition and the EMSs negativism/pessimism, emotional inhibition, unrelenting standards and self-punishment with patient gender

There was a significant association between hypervigilance and inhibition and unrelenting standards (56.52%; p-value <0.0.014) and female gender in patients with migraine (Table 2).

**Table 2:** Evaluation of early maladaptive schemas related to hypervigilance and inhibition according to gender.

Schema		Gender					p-value Chi-square
		Male			Female		
		n	%		n	%	
**Total**		**19/65**			**46/65**		
Negativism/pessimism	>3.8	1/19	5.26		8/46	17.39	0.436
	≤3.8	18/19	94.73		38/46	82.60	
Emotional inhibition	>3.8	1/19	5.26		6/46	13.04	0.654
	≤3.8	18/19	94.73		40/46	86.95	
Unrelenting standards	>3.8	7/19	36.84		26/46	56.52	0.014*
	≤3.8	18/19	94.73		14/46	30.43	
Self-punishment	>3.8	-	-		28/46	60.86	<0.0001*
	≤3.8	18/19	94.73		19/46	41.30	

* Statistically significant

### Association of hypervigilance and inhibition and the EMSs negativism/pessimism, emotional inhibition, unrelenting standards and self-punishment with patient gender and migraine

There was a significant association between hypervigilance and inhibition and unrelenting standards (73.68%; p-value <0.0001) and self-punishment (84.21%; p-value <0.0001) in female patients with migraine (Table 3).

**Table 3:** Evaluation of early maladaptive schemas related to hypervigilance and inhibition according to group.

Schema		Group					p-value Chi-square
		Control			Migraine		
		n	%		N	%	
**Total**		**27/65**			**38/65**		
Negativism/pessimism	>3.8	3/27	11.11		6/38	15.78	0.865
	≤3.8	24/27	88.88		32/38	84.21	
Emotional inhibition	>3.8	1/27	3.70		6/38	15.78	0.301
	≤3.8	26/27	96.29		32/38	84.21	
Unrelenting standards	>3.8	5/27	18.51		28/38	73.68	< 0.0001*
	≤3.8	22/27	81.48		10/38	26.31	
Self-punishment	>3.8	-	-		32/38	84.21	< 0.0001*
	≤3.8	27/27	100		6/38	15.78	

* Statistically significant

## DISCUSSION

This study found a significant association between migraine, female gender, hypervigilance and inhibition, unrelenting standards and self-punishment.

Although the designations of unrelenting standards and self-punishment, which are EMSs linked to the hypervigilance and inhibition domain, were used in this study, it is important to clarify that these findings were not specifically of the EMSs, because they did not meet the requirements established by Young *et al* . 19 on the response screen, as the expected means for this classification should be above an average of 5.0.

During the statistical analysis, it was found that the tendency for a significant result in the 65 subjects evaluated in this study would not indicate a personality disorder related to the EMSs as there were few results with average scores above 5.0. Thus, an average score above 3.8 was chosen for each item on the YSQ-S3 response screen that could characterize at least one psychological profile.^26^

The choice of this mean score was based on reports by other authors^25^^,^ 27 that used the YSQ-S3 in their clinics during workups preceding psychotherapy and suggested that a mean score of 3.0 would be a very basic starting point to understand each patient's way of thinking and the origin of their crib education using the domains.^25^

The unrelenting standards and self-punishment found in this study, for example, demonstrate that these individuals experienced a rigid, repressive education in which there was no possibility of expressing their emotions freely. Thus, individuals who have scores between 3.0 and 5.0 may not have EMSs, but appear to be have a strong tendency to be overly -demanding of himself or herself, thus suggesting a psychological profile or way of being yet without solid evidence of a personality disorder.^28^

Nevertheless, some hold that 'way of being' is related to personality disorders.^26^ Henri Ey regards some people (explosive, theatrical, systematic, meticulous, obsessive, obscene, very emotional and with other difficult traits) as having a pathological ego, characterizing not only a way of being in the world, but above all, a way of existing in the world.^29^

Karl Jaspers affirms that personalities that make people and those around them suffer are not normal. According to Jaspers, abnormal personalities represent non-normal variations of human nature, which can be perfectly understood as personality disorders.^30^ However, 'way of being' for this study was not in this context, because the subjects appeared to affect only themselves with the discomfort of migraine.
